# SNPpy - Database Management for SNP Data from Genome Wide Association Studies

**DOI:** 10.1371/journal.pone.0024982

**Published:** 2011-10-19

**Authors:** Faheem Mitha, Herodotos Herodotou, Nedyalko Borisov, Chen Jiang, Josh Yoder, Kouros Owzar

**Affiliations:** 1 Department of Biostatistics and Bioinformatics, Duke University, Durham, North Carolina, United States of America; 2 Department of Computer Science, Duke University, Durham, North Carolina, United States of America; 3 Department of Biostatistics and Bioinformatics, Duke University, Durham, North Carolina, United States of America; Institut Jacques Monod, France

## Abstract

**Background:**

We describe SNPpy, a hybrid script database system using the Python SQLAlchemy library coupled with the PostgreSQL database to manage genotype data from Genome-Wide Association Studies (GWAS). This system makes it possible to merge study data with HapMap data and merge across studies for meta-analyses, including data filtering based on the values of phenotype and Single-Nucleotide Polymorphism (SNP) data. SNPpy and its dependencies are open source software.

**Results:**

The current version of SNPpy offers utility functions to import genotype and annotation data from two commercial platforms. We use these to import data from two GWAS studies and the HapMap Project. We then export these individual datasets to standard data format files that can be imported into statistical software for downstream analyses.

**Conclusions:**

By leveraging the power of relational databases, SNPpy offers integrated management and manipulation of genotype and phenotype data from GWAS studies. The analysis of these studies requires merging across GWAS datasets as well as patient and marker selection. To this end, SNPpy enables the user to filter the data and output the results as standardized GWAS file formats. It does low level and flexible data validation, including validation of patient data. SNPpy is a practical and extensible solution for investigators who seek to deploy central management of their GWAS data.

## Introduction

Statistical analysis of SNP data from Genome-wide Association Studies (GWAS) typically involves the management and integration of patient information, including phenotypic data, with the genomic SNP data across multiple studies. Issues that have to be addressed include (i) data validation of the patient data and the SNP data, (ii) performance issues with operating on large datasets, and (iii) accurately updating the portions of the data that are rapidly changing, usually the patient data.

Relational databases are a well-known solution to parts of this problem, particularly data validation. However, they have not seen much use in this context, possibly because of performance issues caused by the typically large size of GWAS datasets, coupled with the complex data manipulations the database would need to handle. We show that these obstacles are surmountable, and that a usable and useful system can be built based on a relational database.

A common approach to data management is direct code manipulation of raw data files. For example, directly extracting SNP or patient data from a file, performing any necessary transformations, and writing the results to another file. While this ad-hoc approach is simpler and therefore superficially more attractive, an architected database approach is a scalable and more robust solution. For the remainder of this section, we discuss the advantages of our database solution.

Databases offer considerable and customizable machinery for low level data validation, as described below. This machinery can be used to detect corrupt data. It is particularly important in the case of patient data, which is exceptionally mutable and corruption-prone.

- Column values can be constrained to a fixed set of values defined in auxiliary tables. Our current database schema uses the *allele*, *chromo*, *race*, *sex*, and *snpval* tables for providing data constraints on our main tables by restricting certain columns to the contents of the aforementioned tables (as discussed in the Design Subsection).
- Less restrictively, column values can be confined to a specific type. For instance, a specific column can be configured to only accept integers or character strings of a fixed or minimum or maximum length. Our schema contains many examples of type constraints on integer and character values. For example, the *chromosome* and *location* columns are constrained to be integers.
- A more general category of constraint is *check constraints*, which allows the user to specify that the value in a certain column must satisfy a Boolean (truth-value) expression.
- Other useful constraints are *unique constraints*, which ensure that the data contained in a column or a group of columns is unique with respect to all the rows in the table. Uniqueness is used in a number of places. For example, all main identifiers, like *fid* in the *anno* table, and *name* in the *chromo* table, are constrained to be unique.

These constraints are essential in identifying corrupt data. For example, if a record specifies that a patient's sex is ‘B’ (the only valid sex values are ‘F’ for female and ‘M’ for male), the database will return an error on loading. Similarly, if a string value is specified for a SNP location (only integer values are allowed) the database will return an error. Also, genotype data is converted to and constrained to be stored as integers from the set {−1, 0, 1, 2}, which makes corruption of this data unlikely.

It is extremely valuable to be working with validated data on the outset. In contrast, tools not using a database typically ‘hardwire’ their more limited validation checks. For example, PLINK [1] and GenABEL [2] verify that for each SNP the same number of patients have been called and that the SNPs are bi-allelic. As such tools do not use more fine-grained validation, they must rely on testing ‘downstream’ of the initial data import, at which point the data may have already undergone post-processing, and hence errors may be harder to detect.

**Figure 1:**
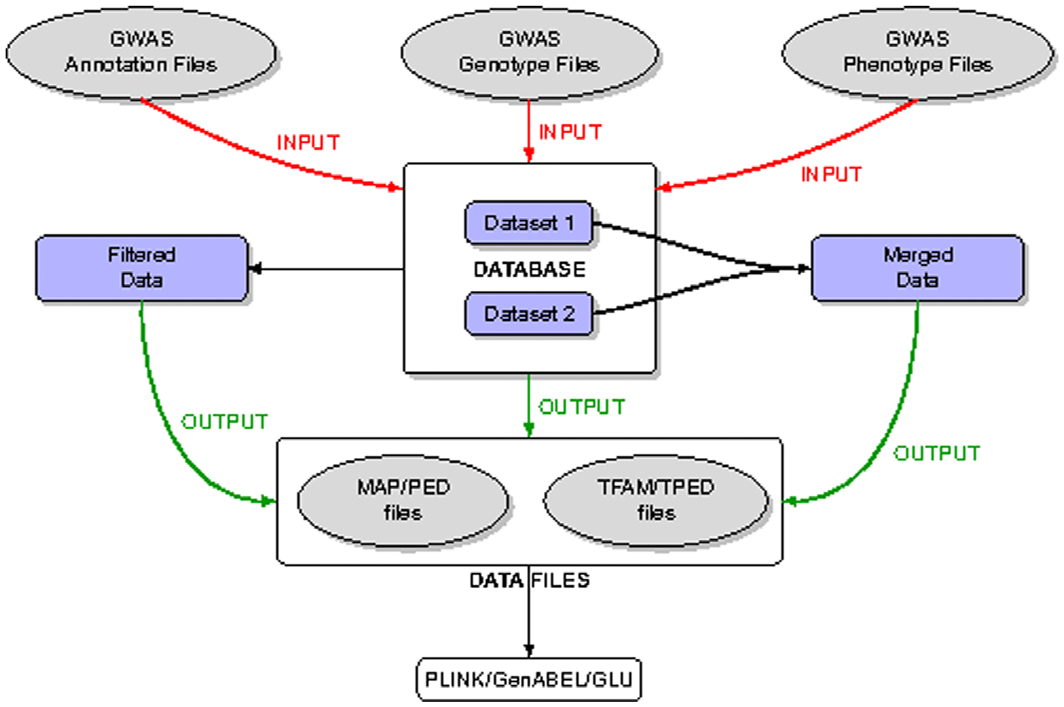
Workflow Chart. This figure shows the data workflow. First the genotypic and phenotypic data are loaded into the database. The data is then exported from the database as standard format files, including a possible filtering and/or merging step. Finally, the output files are further analyzed using third party tools.

Consider the task of converting GWAS data, consisting of phenotype and genotype data, into standard format files like PED/MAP or TPED/TFAM, or merging data across different GWAS datasets. An approach based on manipulating these files requires the software to accommodate different source data formats like those used by Affymetrix and Illumina. Such functionality would thus depend on both the source data format and the output data format, so the number of functions required is of the order of 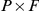, where 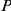 and 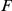 are the number of source formats and the number of output formats respectively. In contrast, our system breaks this task down into two distinct parts. The first part involves *loading* data from various files into the database; a task which is commonly referred to as Extract-Transform-Load (ETL). The second part involves *exporting* data from the relational database via Structured Query Language (SQL) queries to standard format data files for use in further analyses. This process is illustrated in Figure 1. For simplicity, assume one database layout/schema with possible minor variations caused by differences in source formats. Then we can write a single ETL function for the import of each commercial or ad-hoc source format. The database export functions need not depend on the source format, since the database layout is sufficiently independent from the source format. Therefore the data export for each output format can be implemented as a single SQL query. Thus the number of functions required here is of the order of 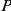
+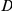, and this simplifies the task.

Databases also allow for repeatable and optimized data transformation tasks to be executed using a standardized and universally accepted language, namely SQL. SQL query execution is the benchmark metric of the Relational Database Management System (RDBMS) industry and as such has been finely optimized in all enterprise class databases. Hence, we use SQL for the task of manipulating and exporting data from the database. The results of the database query can be analyzed externally using statistical software such as PLINK [1], GenABEL [2] or GLU [3], or internally using PostgreSQL plugin pl/R [4].

## Design and Implementation

### Development Environment

Our testing and development environment is a machine with four quad-core AMD Opteron processors (16 cores total) and 64 GB of Random Access Memory (RAM). Our system consists of two major components: a database to store and manipulate the data, and a high-level interface to communicate with it.

PostgreSQL was a clear choice for the database, as it is the leading open source industrial strength database, and is competitive in quality and performance with the major proprietary databases.

Parsing data files requires a high level, preferably interpreted language with good text processing capabilities. Python is a leading open source interpreted language with excellent support for text manipulation and object oriented design. For the communication mechanism between Python and PostgreSQL we use the Python library SQLAlchemy. SQLAlchemy offers more functionality than a database adapter like psycopg2. Among other features, SQLAlchemy includes an Object Relational Mapper (ORM), which enables the use of object oriented programming with a relational database.

### Porting to Alternative Software Environments

It should not be difficult to port the current system to use another suitably advanced RDBMS. The proprietary Oracle database is comparable in features to PostgreSQL and is a possible candidate. However, since Oracle is a proprietary system, the resulting system will be dependent on a proprietary product. SQLAlchemy (which we use for database communication) supports the Oracle database, and the system for the most part uses standard SQL queries. Current versions of Oracle support all the advanced SQL features used, including Common Table Expressions (CTEs) and window functions. Hence, this port should be possible with relatively minor changes. An example of a PostgreSQL specific extension is the COPY command, which is used for bulk loading and exporting of data. This would have to be replaced by a suitable Oracle equivalent, possibly SQL*Loader. There are also some minor uses of PostgreSQL's SQL Procedural Language (PL/pgSQL) which would need to be rewritten.

The other main part of the system is Python scripts which use the SQLAlchemy database interface. Replacing Python with another language would of necessity require replacing SQLAlchemy, which is written in Python. This would be more difficult than replacing the database, since the system depends heavily on SQLAlchemy's capabilities, including both high level (ORM) style functionality as well as low level functionality. Significant rewriting would probably be necessary.

### Design

**Figure 2:**
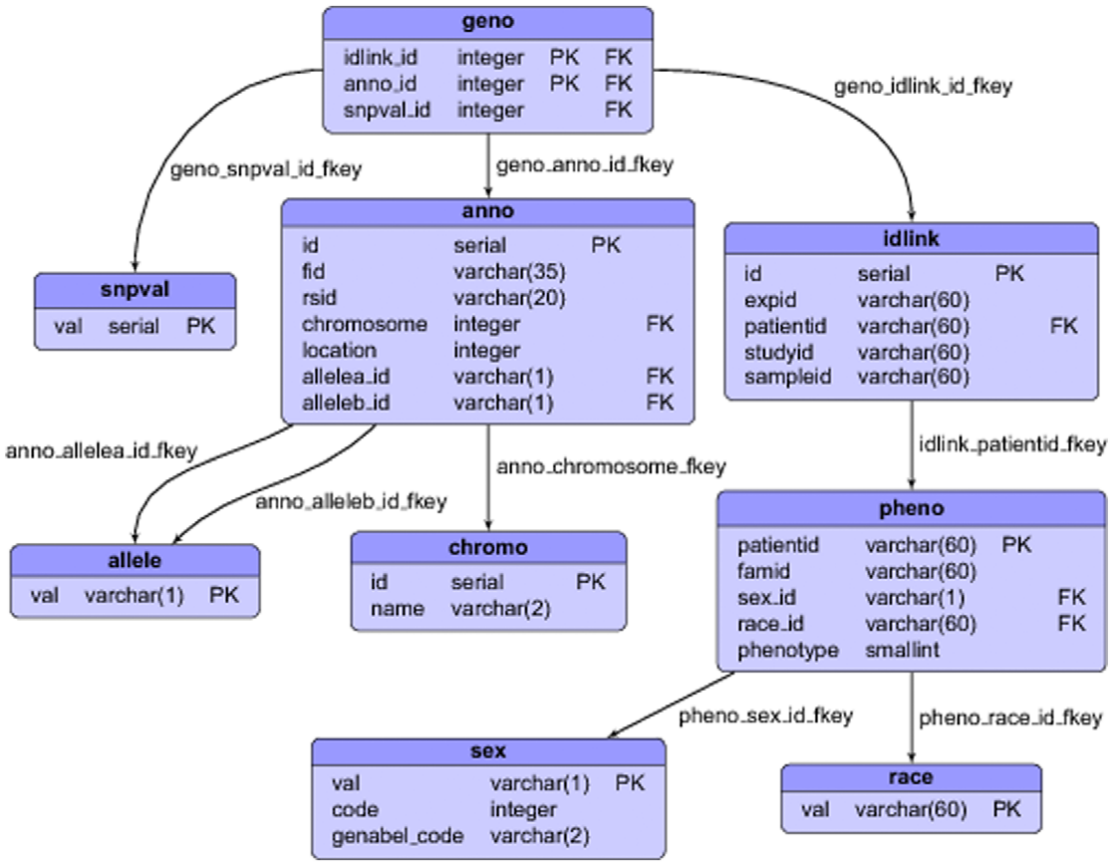
Database Schema. *Geno Single* database schema for the Affymetrix platform. In this diagram, the rectangles correspond to database tables, and the rows in each rectangle correspond to database table columns. The four columns in a row correspond to, from left to right, database name (column 1), data type (column 2), primary key indicator (column 3), and foreign key indicator (column 4). The arrows correspond to foreign keys. Observe the number of arrows leaving a table is equal to the number of columns that are foreign keys in that table.

The heart of SNPpy is the database schema illustrated in Figure 2. In addition to the schema, we have developed two classes of Python scripts: (i) *input scripts* for parsing and loading the database tables, and (ii) *output scripts* for processing and exporting the data into different downstream formats, using SQL queries. The input scripts are written using object-oriented Python, with classes corresponding to the different platforms. Currently, the system can produce PED/MAP and TPED/TFAM data formats for individual datasets as well as the merger of multiple datasets. The latter is useful, for example, for doing quality control with HapMap data. A diagrammatic representation of the overall workflow is shown in Figure 1.

We have two slightly different database layouts, namely *Geno Single* and a variant called *Geno Shard*.

The database schema in the *Geno Single* case consists of nine tables. The *pheno* table contains the phenotypic data, where the table's primary key (the unique identifier) is the patient id, as chosen by the clinical study. The *anno* table contains SNP annotation data, including the chromosome, base-pair position and reference alleles for each SNP. The unique id for this table is the label assigned to the SNPs by the platform manufacturer. The *geno* table contains the genotype calls for the experiments and has a composite primary key consisting of idlink and anno ids. So each entry in this table is uniquely identified by both an idlink and anno id. The *idlink* table contains both experimental id and patientid columns. The former identifies samples, and the latter is a foreign key pointing to the *pheno* table and identifies patients. So this table connects the phenotypic and genotypic information located in the *pheno* and *geno* table respectively. In general, a single patient id may correspond to multiple experimental ids. The data in the *pheno*, *anno* and *geno* tables naturally correspond to the imported phenotype, annotation, and genotype/calls files. We have a number of auxiliary tables, namely *allele*, *chromo*, *race*, *sex*, and *snpval*, whose sole purpose is to provide data constraints on our main tables.

The *Geno Shard* layout is similar to the layout above, with the exception that the genotype data is partitioned into multiple tables, such that rows having the same experimental id are placed in the same table. This creates one genotype table per sample. The intention is to optimize both data loading and exporting to the PED file format.

**Figure 3:**
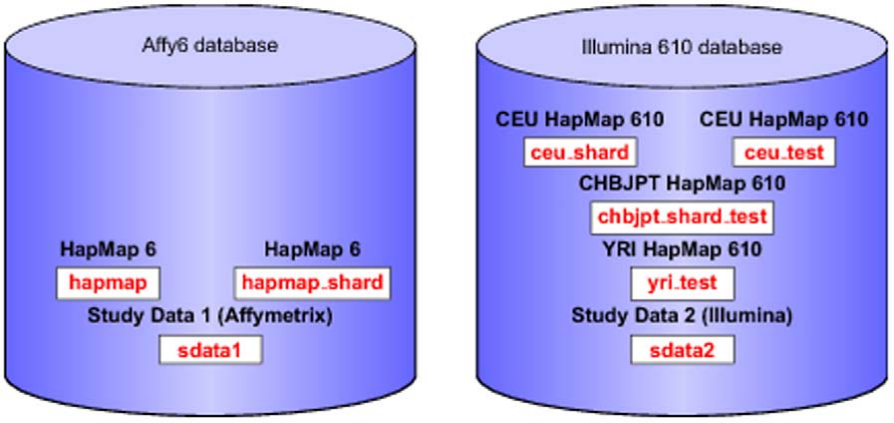
Database Layout. Datasets for different platforms are stored in separate databases, here represented by cylinders. Every dataset is stored in a separate database schema (namespace within a database). The same dataset can be stored in multiple schemas, differing in what options have been selected when loading the dataset. To illustrate this, the figure shows the schemas in red and the datasets in black. Each of the datasets *HapMap 6* and *CEU HapMap 610* is stored in two schemas. For further details see the manual.

We currently support two genotyping platforms, Affymetrix and Illumina. We use both the database layouts described above for each platform. We use one database for each platform. Within each database, each schema corresponds to a dataset (PostgreSQL's namespace within a database). Figure 3 provides a graphical representation of the data architecture.

### Performance and Optimization

First, we consider database loading. For the *Geno Single* layout, given 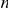 patients and 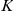 SNPs, the resulting database tables *anno* and *geno* will be of sizes 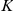 and 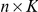 respectively. For the *Geno Shard* layout, instead of one *geno* table, we have 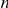 genotype tables of size 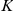 each.

The genotype data accounts for the largest part of the dataset by far. For database tables of this size, naive loading of the database tables, as well as query execution will be unacceptably slow. We use several techniques to optimize these procedures.

Database loading can proceed by loading one row into a table at a time, or by bulk insert of rows. We use the former method for the smaller tables (e.g., *pheno*) with the help of SQLAlchemy's ORM. This method is less efficient but more flexible, and not database specific. We need such flexibility to process the more complex and changeable patient phenotypic data. We use the latter method for the larger tables, e.g. *anno* and *geno* (in the Geno Single case). This method is more efficient, but less flexible, and database specific. Specifically, PostgreSQL offers the low-level COPY command which reads a file into a database table. This file is typically in Comma-separated Values (CSV) format, with the table data laid out in the form of one line per table row. The code that writes this file is in C++ to improve speed, as this process is CPU intensive. We remove the database constraints and indices from the tables before running COPY, in order to prevent PostgreSQL from checking the constraints and updating the indices while the COPY is proceeding. The constraints are checked and indices are restored after COPY completes loading the data into the database.

We now make some specific remarks about the *Geno Shard* layout. This layout was devised to optimize PED file export, and corresponds to one genotype table per sample. The problem of loading these genotype tables is embarrassingly parallel, so we use the Python multiprocessing library to create a pool of processes to load these tables in parallel. As shown in the Experimental Evaluation Subsection, both dataset load times and PED export times are greatly improved by the combination of the *Geno Shard* layout and parallelization. Additionally, the *Geno Shard* routines use less memory than the *Geno Single* versions.

Next, we consider exporting from the database. We employ standard techniques for optimizing SQL queries, specifically advanced SQL features such as Common Table Expressions (CTE) and window functions.

First we address the *Geno Single* case. We use a pool of threads to parallelize the creation of the PED and TPED files in the *Geno Single* case. This requires the Python thread library. Each thread executes multiple SQL queries. Each query creates a different temporary file containing a part of the PED or TPED file contents. Once all the parts have been written, the file is assembled from them.

Now we consider the *Geno Shard* case. Each table in the *Geno Shard* layout contains exactly the genotype data of a single sample. Also, the genotype data in a line of a PED file is exactly that of a single sample. Therefore, to write any line of the file, it is necessary and sufficient to retrieve all the data from a single table. Retrieving all the data from a single table is fast. Therefore, writing PED files in this case is fast, and scales well with increasing data size, since the computational requirements of retrieving data from 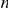 tables scale linearly with 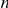. Like *Geno Shard* loading, this is an embarrassingly parallel problem. As with *Geno Single* export, we use a pool of threads to parallelize the creation of the PED file. Here each SQL query retrieves all the data from a single table, and writes a line of the PED file to a temporary file.

## Results and Discussion

### Functionality

SNPpy currently supports two genotyping platforms (Affymetrix and Illumina) and two database layouts (*Geno Single* and *Geno Shard*) for each of these platforms. It can load datasets into the database in these layouts, using the load_dataset.py script. It can export data as MAP, PED, TPED and TFAM data files for the *Geno Single* layout, and as MAP and PED files for the *Geno Shard* layout. For many researchers, the ability to export the data into these standard data formats for use by statistical software such as PLINK, is sufficient. The user can specify a filter condition as a SQL expression in terms of the columns of one of the *anno*, *idlink* and *pheno* tables. Up to three simultaneous filtering conditions are possible, corresponding to the three tables. These conditions restrict the data export to the selected subset. For instance, the user might export data corresponding to selected chromosomes of Caucasian male patients. Furthermore, it is possible to export data files corresponding to merged datasets. All exporting functionality is performed by the make_output.py script. Additionally, SNPpy can update the *pheno* table with an updated pheno data file by using the script update_pheno.py. The above functionality can easily be extended to other platforms and other data formats as outlined below.

There are minor differences between the Affy6 and Illumina platform database layouts. These are restricted to the *anno* table. Similarly, a new platform might require additional changes to the existing layouts. The layouts are all described in dbschema.py using SQLAlchemy, so additional tables would need to be described there.

The platform specific sections of the loading code are in anno.py and geno.py. anno.py contains the classes *Anno_Affy6* and *Anno_Illumina*, which specify the platform specific code to generate the *anno* table. Similarly, geno.py contains classes which have platform specific code for generating the *geno* table. These two files contain all the platform-specific loading code. To add loading support for another platform, similar classes would need to be written.

The export functions are in output.py. For a given database layout there is one function to export the data to a given format. In most cases, this is a single SQL statement. The variations in SNPpy database layouts are mostly due to the two database layouts *Geno Single* and *Geno Shard*. As already mentioned, layout differences between the Affy6 and Illumina platforms are minor. So, currently there are two functions for every supported export format, one each for *Geno Single* and *Geno Shard*. The upshot is that a new export format can be added by writing suitable functions in output.py.

SNPpy uses a single configuration file to control the annotation, import and export of multiple studies. We expect this feature to significantly simplify project management for researchers dealing with multiple studies.

Additionally, SNPpy provides a script, simdat.py, to generate simulated phenotype and genotype data, and a test script, test_simdata.py, which currently tests SNPpy code functionality for both Affymetrix and Illumina platforms using simulated phenotype and genotype data. The simulated data is useful for testing SNPpy performance for large data sets, and tuning the PostgreSQL configuration for better performance. The test script is useful for quickly checking whether SNPpy runs in a given environment.

For usage details, see the file docs/MANUAL in the source repository.

### Experimental Evaluation

For testing purposes, we applied SNPpy to two GWAS studies, one consisting of 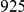 patients and typed on the Affymetrix SNP 6 chip, the other consisting of 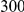 patients and typed on the Illumina Human 610-Quad chip. In what follows we refer to these as Study Data 1 and 2 respectively. Additionally, we tested SNPpy on simulated Affymetrix SNP 6 and Illumina Human 610-Quad datasets of varying sizes.

In our testing, we chose simulated datasets of sizes comparable to the size of the 1958 British Birth Cohort dataset referenced on the Wellcome Trust Case Control Consortium website [5]. The number of SNPs processed was 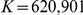 for the Illumina and 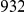,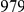 for the Affymetrix platform. A standard quality control check for GWAS data consists of comparing genetic ancestry based on the study SNP data to self-reported ancestry and to genetic ancestry estimated from the HapMap samples for that platform. To this end, the study and HapMap data need to be merged. For each platform, we obtained a set of HapMap samples from the project webpage [6], which we merged with the respective study data. For Study Data 1 (Affymetrix SNP 6), we preprocessed 901 CEU, CHBJPT and YRI CEL files into a single genotype calls file (denoted as HapMap 6). CEU denotes Utah residents with Northern and Western European ancestry from the Centre d'Etude du Polymorphisme Humain (CEPH) collection. CHB denotes Han Chinese in Beijing, China. JPT denotes Japanese in Tokyo, Japan. YRI denotes Yoruba in Ibadan, Nigeria. For Study Data 2 (Illumina Human 610-Quad), we obtained separate preprocessed HapMap call files for CEU, CHBJPT and YRI of sizes 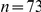, 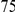 and 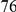 respectively.

**Figure 4:**
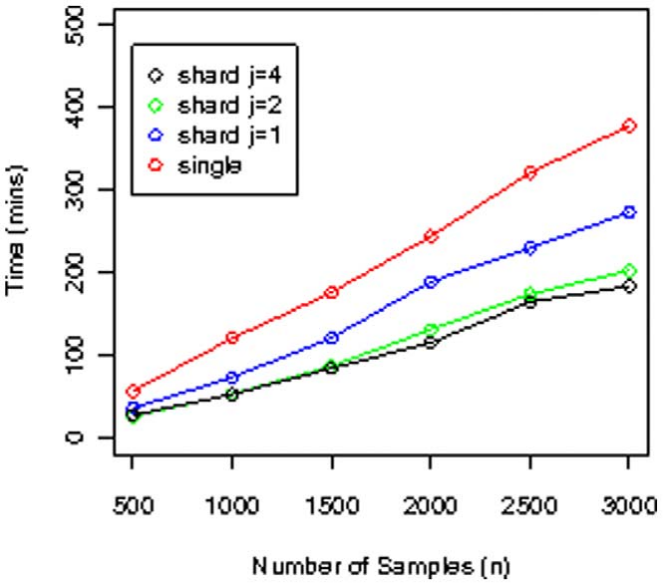
Dataset load timings. Timings for loading simulated datasets for the Illumina platform into the database, for the *Geno Single* layout, and the *Geno Shard* layout with degree of parallelism 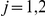 and 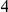. For all these datasets, the number of SNPs is 620,901.

**Figure 5:**
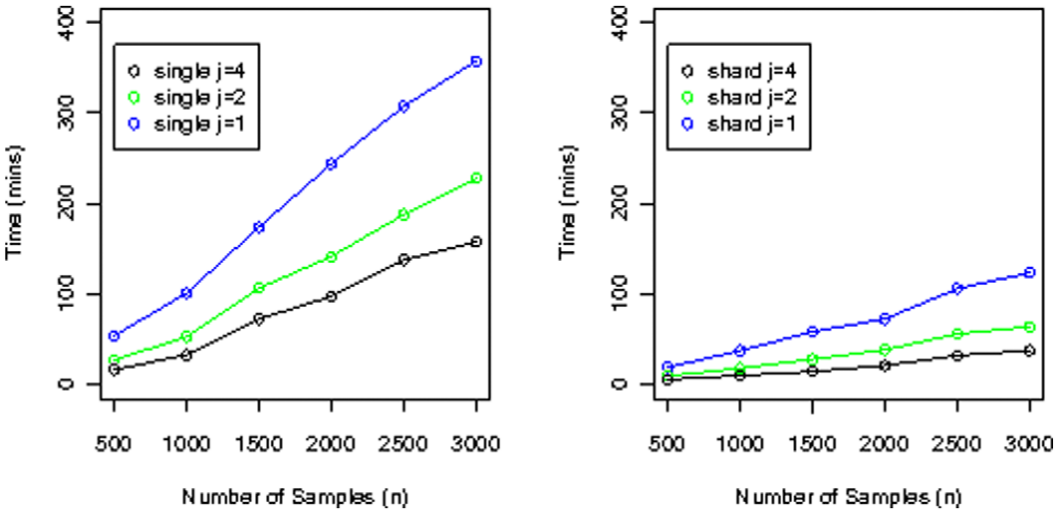
PED file write timings. Timings for writing PED files from simulated datasets for the Illumina platform, for the *Geno Single* layout with degrees of parallelism 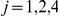 and *Geno Shard* layout with degree of parallelism 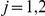 and 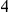. For all these datasets, the number of SNPs is 620,901. All timings correspond to warm cache.

**Figure 6:**
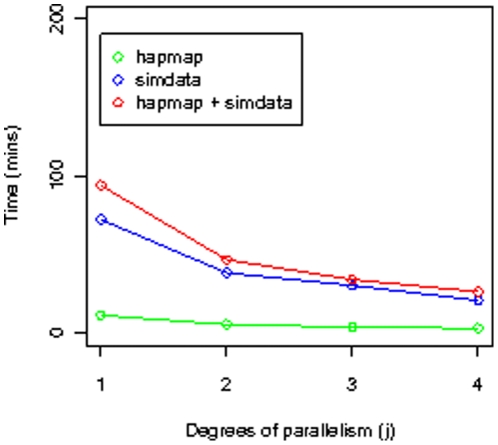
PED file merged write timings. Timing results for writing the PED file corresponding to the merger of the 2000 patient Illumina simulated dataset with the corresponding HapMap datasets compared to timings for writing the PED file for each of the 2,000 patient simulated dataset and the Hapmap dataset. All these timings are for the *Geno Shard* layout. For all these datasets, the number of SNPs is 620,901. All timings correspond to warm cache.

Import and export (into PED format) timings for simulated Illumina datasets are shown in Figures 4 and 5. All timings correspond to warm cache (the query was run before timings were taken, so the data is already in memory). Timing results for merging simulated Illumina datasets with the corresponding HapMap datasets are shown in Figure 6.

### Comparisons with Other Software

Two software packages that utilize a database backend for managing SNP and phenotype data are GWAS Analyzer [7] and SNPLims [8].

The SNPLims authors provide PED export timing for an Illumina HumanHapMap300 (317 K SNPs) dataset with 100 samples. They report a processing time of 10 minutes. The results may not be directly comparable, as the SNPLims authors used different hardware (Intel Xeon 2.4 Ghz processor with 1 G of RAM) and software (PostgreSQL 8.1). As the SNPLims code is not available, we were unable to conduct a more comprehensive comparison.

We downloaded GWAS Analyzer from http://www.nwrce.org/gwas-analyzer. The authors of GWAS Analyzer do not report performance metrics in the large scale setting. They conjecture (Section 4.2 in [7]) that their system should scale to larger studies by using more processing power, memory and storage space. We measured import/export timings on GWAS Analyzer using the same server that was used for reporting performance metrics of SNPpy, and with a data set of the same size and type as used by SNPLims, namely an Illumina HumanHapMap300 (317 K SNPs) dataset with 100 samples. We also measured timings using the SNPpy *Geno Single* layout, and the *Geno Shard* layout with 4 parallel database processes (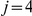). The time taken for loading the dataset was 18 minutes for GWAS Analyzer, compared to 4.2 minutes using *Geno Single*, and 2.6 minutes using *Geno Shard*. The corresponding MAP and PED export times were 93 minutes using GWAS Analyzer, compared to 5 minutes using *Geno Single*, and 0.75 minutes using *Geno Shard*. See Table 1 for the comparison timing summary. The processing times of both SNPpy and GWAS Analyzer were measured with the UNIX time command, and as before they were warm cache timings.

**Table 1:** Software timing comparisons.

	SNPLims	GWASA	SNPpy	
			Geno Single	Geno Shard ( 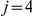 )
loading time	-	18	4.2	2.6
writing time	10	93	5	0.75

Sample software timing comparisons for SNPpy, SNPLims and GWAS Analyzer (GWASA) for importing and exporting (in MAP and PED format) a Illumina HumanHapMap300 (317 K SNPs) dataset with 100 samples. The times are measured in minutes using the UNIX time command.

GWAS Analyzer and SNPLims currently provide web interfaces to their respective systems, and support other genotypic and annotation information such as the call rate, Illumina *GeneCall* score, and mutation type. While the extensible design of SNPpy enables adding similar functionality, its current focus is on efficiency, as evinced by the timing comparisons above, and flexibility. Its database loading uses a variety of techniques, including the customized database layout of *Geno Shard*, multithreading, and multiprocessing. SNPpy exports data using a single SQL query per job in most cases. In contrast, the export facility in GWAS Analyzer is a Perl script, which uses many SQL queries and also manipulates the data using Perl, incurring a performance penalty. Additionally, SNPpy has more features than GWAS Analyzer for both import and export. Some of the additional features provided by SNPpy include:

- Use of a centralized configuration system.
- Separation of datasets into different namespaces (PostgreSQL schemas).
- Storage of the genotype calls as 0, 1, 2, −1 instead of letter pairs.
- Support for both Affymetrix and Illumina platforms (can easily be extended to other platforms).
- Support for loading a subset of the data corresponding to a subset of the SNPs.
- Support for exporting to transposed filesets (TPED/TFAM).
- Support for data set filtering.
- Support for data set mergers.
- Native translation of stranding format. Currently, SNPpy supports TOP and FORWARD encoding for Illumina. Other encodings could be added.

## Availability and Future Directions

### Future Development

Currently, SNPpy can import data from upstream source data files. A feature to import data from standard data format files like MAP and PED could also be added.

The current version of SNPpy assumes a single outcome phenotype of type *smallint*. For a GWAS conducted as a correlate to a clinical trial, the relevant phenotype data are, compared to a typical case-control study, invariably more complex, consisting of large numbers of qualitative and quantitative demographic and clinical variables. SNPpy can be extended to include these complex data sets. This can be accomplished by importing the relevant data as a database table, which can then be queried against when conducting export queries. The resulting data can be stored in a text file to be used by statistical software for downstream analysis. Specifically, both PLINK and GenABEL import additional phenotypes through text files paired with the PED/MAP (or TPED/TFAM) files.

A natural next step after importing genomic data into PostgreSQL is to employ procedural languages for PostgreSQL such as pl/R [4] to carry out the analyses directly on the database and export the statistical results rather than the data. The major advantage of this approach as opposed to usual R usage is that R loads all data into memory, while a database does not. Therefore, the database approach would be faster, use less memory, and support data sizes that do not fit in memory.

While storing genotype calls from next generation whole-genome scans into a database system may not be practical due to the enormous size of the resulting data, SNPpy could be readily used to store the subset of SNPs from these scans that are to be used for statistical analyses. For instance, these could be SNPs that have *a priori* been determined to be “functional” according to information from bioinformatics databases.

SNP imputation [9] methods are commonly employed to conduct inference on the basis of SNPs not typed on the GWAS platform. Three commonly used SNP imputation algorithms are IMPUTE [10], [11], BEAGLE [12], [13] and MaCH [14], [15]. As the process of imputation across the entire genome is computationally prohibitive, the task is commonly split up across the chromosomes or other sub-regions of the genome. Accordingly, the study data has to be split into a set of individual files, each restricted to the set of SNPs in the corresponding chromosome. Our proposed database framework can be readily extended to facilitate the requisite preprocessing to produce these files. Given that SNPpy is a Python program, it can be further extended to directly call the imputation program after the files have been generated. Moreover, as the process of conducting imputation analyses across mutually exclusive regions presents an embarrassingly parallel problem, one can readily use multiprocessing, as SNPpy already does for import and export.

### Availability and requirements

- Project name: SNPpy
- Project home page: http://bitbucket.org/faheem/snppy. Please submit bugs to the Bitbucket issue tracker at that page. The mailing list is located at http://groups.google.com/group/snppy. A secondary project home page is http://code.google.com/p/snppy-code/. Please use this in case of problems with Bitbucket. However, Bitbucket should still be considered the main home page.
- Source Code Archive: See File S1.
- Operating system(s): Linux i386 and AMD64. Tested on the following distributions: Debian 5.0 (lenny) and 6.0 (squeeze), Fedora Core 13 (Goddard) and 14 (Laughlin), Ubuntu 10.04.1 LTS (Lucid Lynx), OpenSUSE 11.3.
- Programming languages: Python (2.6 or later), SQL, C++.
- Other requirements: SCons, PostgreSQL (8.4 or later), SQLAlchemy (0.5.× or 0.6.×), Python ConfigObj library (4.5.2 or later), psycopg2 (2.0.7 or later), Boost C++ libraries.
- License: GNU General Public License (GPL), version 2 or later.

### Conclusions

We have described a hybrid script database system to comprehensively manage genotype and phenotype data from multiple genome-wide association studies. The current version provides facilities for importing SNP data from two major commercial platforms, and exporting filtered data in two standard formats. Comparisons of processing times with those of two other published systems that use database backends to manage GWAS data show SNPpy has considerably faster processing times. The system can be readily extended to import data from other platforms by adding custom loading functions for the genotype call and annotation data. The database layout can be optimized for specific types of exports. We have developed such a database layout, i.e. *Geno Shard*, for PED exports. In summary, SNPpy provides a practical and flexible framework for researchers seeking to be able to manage and query their GWAS data in a systematic and flexible fashion.

## Supporting Information

File S1 **Source code.** SNPpy source code in an archive file format. See docs/MANUAL for usage information. This archive corresponds to a revision of the Mercurial repository. That revision can be identified by the character string which follows the *snppy* string in the archive name. That string is the revisions's hash identifier (short version).
(TBZ2)
